# Morpho-Molecular Characterization of Two *Ampelomyce*s spp. (Pleosporales) Strains Mycoparasites of Powdery Mildew of *Hevea brasiliensis*

**DOI:** 10.3389/fmicb.2018.00012

**Published:** 2018-01-19

**Authors:** Kapila K. Liyanage, Sehroon Khan, Siraprapa Brooks, Peter E. Mortimer, Samantha C. Karunarathna, Jianchu Xu, Kevin D. Hyde

**Affiliations:** ^1^Center of Excellence in Fungal Research, and School of Science, Mae Fah Luang University, Chiang Rai, Thailand; ^2^Center for Mountain Ecosystem Studies, Kunming Institute of Botany, Chinese Academy of Sciences, Kunming, China; ^3^World Agroforestry Centre, East and Central Asia, Kunming, China; ^4^Rubber Research Institute of Sri Lanka, Agalawatta, Sri Lanka

**Keywords:** *Erysiphe*, haplotype, morphology, mycohost, mycoparasite

## Abstract

Powdery mildew disease of rubber affects immature green leaves, buds, inflorescences, and other immature tissues of rubber trees, resulting in up to 45% losses in rubber latex yield worldwide. The disease is often controlled by dusting the diseased plants with powdered sulfur, which can have long-term negative effects on the environment. Therefore, it is necessary to search for alternative and environmentally friendly control methods for this disease. This study aimed to identify mycoparasites associated with rubber powdery mildew species, and characterize them on the basis of morpho-molecular characteristics and phylogenetic analyses of ITS rDNA regions. We observed that the *Ampelomyces* fungus parasitizes rubber powdery mildew, and eventually destroys it. Furthermore, on the basis of phylogenetic analyses and morphological characteristics we confirmed that the *Ampelomyces* mycoparasite isolated from rubber powdery mildew is closely related to other mycohost taxa in the *Erysiphe* genus. A total of 73 (71 retrieved from GenBank and two obtained from fresh collections of rubber powdery mildew fungi) *Ampelomyces* spp. were analyzed using ITS rDNA sequences and 153 polymorphic sites were identified through haplotypic analyses. A total of 28 haplotypes (H1–H28) were identified to have a complex network of mutation events. The results from phylogenetic tree constructed on the basis of maximum likelihood analyses, and the haplotype network tree revealed similar relationships of clustering pattern. This work presents the first report on morpho-molecular characterization of *Ampelomyces* species that are mycoparasites of powdery mildew of *Hevea brasiliensis*.

## Introduction

Powdery mildew disease devastates rubber [*Hevea brasiliensis* (Willd. Ex A. Juss.) Müll. Arg.] harvests worldwide (Beeley, 1933; Mitra and Mehta, 1938; Limkaisang et al., 2005). Infection results in a secondary leaf fall of the young leaves that emerged after wintering (Fernando, 1971; Min et al., 2012). The causal agent of powdery mildew disease of rubber trees was first described as *Oidium heveae* by Steinmann (1925). Later, Limkaisang et al. (2005), Takamatsu et al. (2007), and Liyanage et al. (2017) discovered that plurivorous *Erysiphe quercicola* (S. Takam. and U. Braun), which was known to cause powdery mildew in several other tree species, also infects rubber plants. Powdery mildew disease can result in the loss of up to 45% of latex yields, reduced girthing-up of the young rubber trees, and slower bark renewal after harvesting (Fernando, 1971; Min et al., 2012; Liyanage et al., 2016). Furthermore, according to Fernando (1971), the flowers of the rubber tree are especially susceptible to powdery mildew, and infection can damage the growth of fruit pods and cause seed loss of up to 90–100%.

Currently, chemical application is the main method to control this rubber tree disease and most commonly used fungicide is sulfur dust (Murray, 1930; Lim, 1982; De S Liyanage and Kuruvilla, 1992; Jayasinghe and Jayaratne, 1997; Tang, 2005). During the process of sulfur dusting, disease control is achieved by blowing powdered sulfur into the rubber tree canopy four to seven times at intervals of 7 days (De S Liyanage and Kuruvilla, 1992; Jayasinghe and Jayaratne, 1997; Priyadarshan et al., 2005). Sulfur does not kill the fungus on the leaf, but protects the leaf from fresh infections (Jayasinghe, 2001). Therefore, the mycelium already on the leaf continues its life cycle, and frequent applications at precise intervals are necessary for effective control of the disease. Applying sulfur dust is expensive, labor intensive, environmentally hazardous and time-consuming, and enormous quantities of sulfur are required worldwide every year (De S Liyanage and Kuruvilla, 1992). The cost of sulfur and other chemical fungicides, and their long-term effects on the environment and human health, necessitate the development of alternative methods for controlling powdery mildew disease.

Biological control agents (BCAs) have the potential to provide alternative methods of preventing or suppressing powdery mildew in some crops (Kiss, 2003). Bio-fungicidal products such as AQ10 Biofungicide^®^, Q-fect, Powdercare^®^, and Sporodex^®^ have been registered and are available in some countries (Kiss, 2003; Park et al., 2010). Several antagonistic fungi from different families have been tested against powdery mildew in other crops, but not yet in rubber plantations. An application of aqueous solutions of *Acremonium byssoides* W. Gams and T. M. Lim has been shown to control powdery mildew disease on rubber trees. However, very little is known about the natural occurrence and activity of *A. byssoides* on rubber trees (Kiss, 2003; Liyanage et al., 2016). *Ampelomyces quisqualis* Ces. is a well-known parasite of powdery mildews which is widely distributed throughout the world. *A. quisqualis* is able to penetrate the hyphae of powdery mildew fungi growing internally from cell to cell through the septal pores. *Ampelomyces quisqualis* then produces pycnidia (fruiting bodies) intracellularly in the hyphae, conidiophores and immature ascomata of their mycohosts, inhibiting conidial production and cleistothecial (closed fruiting bodies of powdery mildews) development (Emmons, 1930; Speer, 1978; Hashioka and Nakai, 1980; Kiss et al., 2004). The pycnidia help to the fungus to overwinter and produce spores. In terms of long-term exposure, the spores of mycoparasites are unlikely to remain viable for long periods of time. Thus, for successful germination, the spores need both favorable conditions (high humidity or moisture and temperatures of 25°C) and the presence of the appropriate host. Without the host, viability is lost within a few days (Mhaskar and Rao, 1974; Szentiványi and Kiss, 2003; Kiss, 2008; Angeli et al., 2011). However, Jarvis and Slingsby (1997), and Angeli et al. (2011) pointed out that *A. quisqualis* can survive and infective to powdery mildews even at temperatures below 12°C. Although genetic diversity of *Ampelomyces* spp. in different mycohost groups has been formerly documented, the single name *A. quisqualis* is still applied to all pycnidial fungi hyperparasites of powdery mildew (Mhaskar and Rao, 1974; Belsare et al., 1980; Kiss and Vajna, 1995; Kiss, 1997). Park et al. (2010) conducted a phylogenetic study using 254 *A. quisqualis* isolates, and showed that *Ampelomyces* hyperparasites are indeed an assemblage of several distinct lineages rather than a single species. The correlation between *Ampelomyces* isolates and their mycohosts is not clear. Although, they do not show exact host specificity, most of the *Ampelomyces* isolates showed adaptation to their mycohosts during the evaluation as a hyperparasite. Also, Kiss (1997), Kiss and Nakasone (1998), and Szentiványi et al. (2005) showed that *Ampelomyces* isolates were mainly divided into two separate clades, each of which was characterized by fast or slow growing in isolated cultures. These authors imply that fast growing *Ampelomyces* isolates should be re-classified as *Phoma glomerata* (Corda) Wollenw. and Hochapfel based on the results of molecular analysis and differences in pycnidia formation (Sullivan and White, 2000; Szentiványi et al., 2005). In recent classification by Chen et al. (2015) *Phoma glomerata* was grouped under the genus Didymellaceae and renamed as *Didymella glomerata* (Corda) Qian Chen and L. Cai.

Therefore, this study aims to clarify the taxonomic positions of *Ampelomyces* mycoparasitic fungi through morphological examination, morpho-molecular, and phylogenetic analyses. This work will be a ground base for future work in developing BCAs against powdery mildew disease of rubber trees.

## Materials and Methods

### Sample Collection

Leaves, buds and the inflorescences of the rubber tree infected with powdery mildew were collected from different major rubber growing areas in Jinghong, Yunnan, China (21°59′52.54′′N and 100°46′17.17′′E), Matugama, Nivitigalakele, Sri Lanka (6°30′44.35′′N and 80°7′5.04′′E), and Muang, Chiang Rai, Thailand (20°2′43.98′′N and 99°53′34.63′′E). Three samples per tree were collected from 10 trees at five different sites where the occurrence of disease was very high from each country discussed above. However, only leaf samples were used for further research. Fresh materials were wrapped in a sterilized moist paper towel, sealed in zip-lock plastic bags, and stored at 5°C soon after returning to the laboratory. The *Ampelomyces* spp. infected leaves collected from China were incubated in plant growth chamber for 2 weeks at 25 ± 2°C (70% RH) under 8:16 dark to light ratio. The growth of *Ampelomyces* spp. and the devastation of the powdery mildew of rubber were photographed every day for time period of 2 weeks.

### Morphological Examination

Surface structures from leaves were stripped off using double-sided clear adhesive tape as described by Cook et al. (1997), and mounted with the specimen’s surface uppermost in an aqueous potassium hydroxide solution (3%) for examination under a compound microscope. Pycnidia were examined by simply pressing a slide against an infected leaf and by simply sticking an infected leaf to a slide with double-sided adhesive tape. Melzer’s reagent (chloral hydrate 100 g, potassium iodide 5 g, iodine 1 ± 0.5 g, distilled water 100 ml) was used as a mounting medium for dried material. Photographs were taken by a Canon digital camera fitted to a Nikon 80i compound microscope. Micro-morphological data such as the size and shape of the pycnidia, conidia, and other micro-structures were recorded, along with the growth habits of the mycelium. All macro-morphological observations and photographs were carried out at different magnifications such as 200×, 400×, and 1000×. Measurements were made with the Tarosoft (R) Image Frame Work program, and the images were processed with Adobe Photoshop software (Adobe Systems, Inc., United States). Pycnidia of *Ampelomyces* species were isolated from powdery mildew infected rubber tree leaves by transferring them with glass needles into Petri plates containing Potato Dextrose Agar (PDA) (20 g dextrose, 15 g agar, and 4 g potato starch) supplemented with 2% malt extract and 0.5% chloramphenicol (Sigma–Aldrich, St. Louis, MO, United States), and cultured at 25°C using a 12-hour cycle of fluorescent illumination.

To observe the surface features at higher magnification on fresh material, 0.5 cm squares were cut out of the leaf lamina and mounted for cryomicroscopy in the SEM. The fragments were mounted on aluminum stubs with double-sided adhesive tape, coated with gold palladium, and then observed under a SEM (Hitachi S4800) (Cook et al., 1997). All micro-morphological observations were carried out at magnifications of up to 12,000× level.

### Fungal DNA Extraction, PCR Amplification, and Sequencing

For the fungal molecular study, ITS regions of rRNA genes were sequenced from the pycnidia of fungal isolates collected from China. Total DNA was extracted from the mycelia by the Chelex method (Walsh et al., 1991; Hirata and Takamatsu, 1996; Limkaisang et al., 2005). The PCR was performed in a Thermal Cycler in a total volume of 25 μl. The PCR mixtures contained Taq DNA polymerase 0.15 μl, 2.5 μl of 2× PCR buffer with 2.5 μl of dNTPs, 1.6 μl of 25 mM MgCl_2_, 1 μl of each primer, 50–100 ng of DNA template and the balance amount of double-distilled water. The genomic rDNA ITS region was amplified by using primers ITS5 (5′-GGAAGTAAAAGTCGTAACAAGG-3′) and P3 (5′-CTATTGCTTGATTGTCTCC-3′) and both strands of the amplicons were sequenced by using the primers ITS1, ITS4, ITS5, and P3 (White et al., 1990; Kusaba and Tsuge, 1995; Hirata and Takamatsu, 1996). The PCR amplicons were electrophoresed on 1.5% agarose gels in TAE buffer. The desire bands were purified from the gel, and amplified fragments were sent to a commercial sequencing provider (Tao Yang, Beijing, China). The nucleotide sequence data acquired were deposited in GenBank.

### Phylogenetic Analyses

The closest taxa to our samples were determined with standard nucleotide blast searches against the nucleotide database in GenBank^1^. Analysis of ITS in the closest taxa in the genus *Ampelomyces* was used to confirm the phylogenetic placement within the genus. The *Phoma* sp. (GenBank Accession number AY663825) was selected as the outgroup taxon based on Park et al. (2010). Multiple sequence alignments were generated with MAFFT v. 7.036 (Katoh and Standley, 2013), and adjusted manually using BioEdit v. 7.2 (Hall, 1999) and ClustalX (Kohli and Bachhawat, 2003). The maximum-likelihood (ML) analysis was performed in RAxML (Stamatakis, 2006) implemented in raxmlGUI v.0.9b2 (Silvestro and Michalak, 2012). Maximum parsimony (MP) analysis was carried out using PAUP (Phylogenetic Analysis Using Parsimony) v. 4.0b10 (Swofford, 2002). Descriptive tree statistics for parsimony (Tree Length [TL], Consistency Index [CI], Retention Index [RI], Relative Consistency Index [RC], and Homoplasy Index [HI]) were calculated for trees generated under different optimality criteria. The Kishino–Hasegawa tests (Kishino and Hasegawa, 1989) were performed to determine whether the trees inferred under different optimality criteria were meaningfully different.

### Haplotypes Identification

The rRNA ITS sequences were assembled and edited in Sequencher 5.0 (Gene Codes, Co., United States). Sequences were aligned with the MUSCLE algorithm (Edgar, 2004). The haplotype of ITS sequences was defined using DnaSP v5.10 (Librado and Rozas, 2009), and the network tree of haplotypes was generated by Network v4.6.1.3 (Fluxus Technology, Ltd.) with the median–joining method (Bandelt et al., 1999).

## Results

### Morphological Study

**FIGURE 1:**
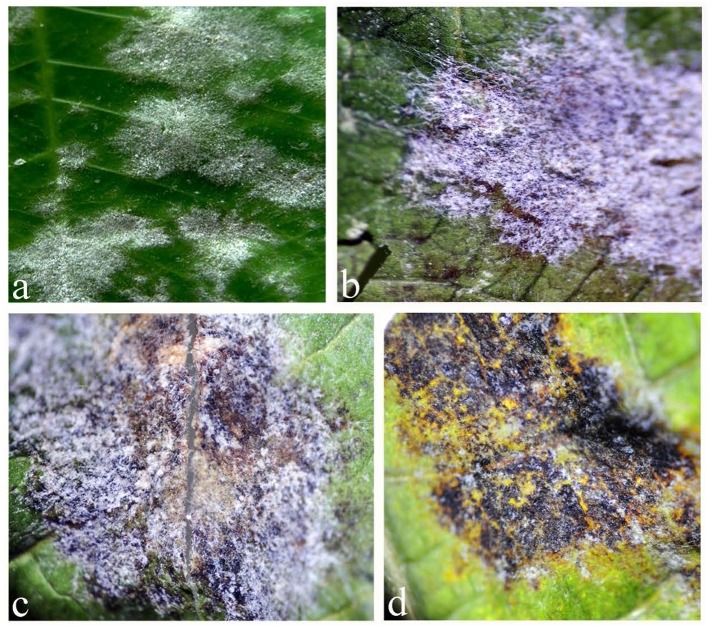
Different stages of *Ampelomyces* spp. infection of powdery mildew fungal colonies: **(a)** healthy colonies of powdery mildew on the surface of rubber leaf; **(b)** powdery mildew colonies infected with *Ampelomyces* spp. (the brown color spots are the pycnidia produced by *Ampelomyces*); **(c)** powdery mildew colonies partially destroyed by *Ampelomyces* spp.; **(d)**. powdery mildew colonies totally destroyed by *Ampelomyces*, 1–2 weeks after infection.

**FIGURE 2:**
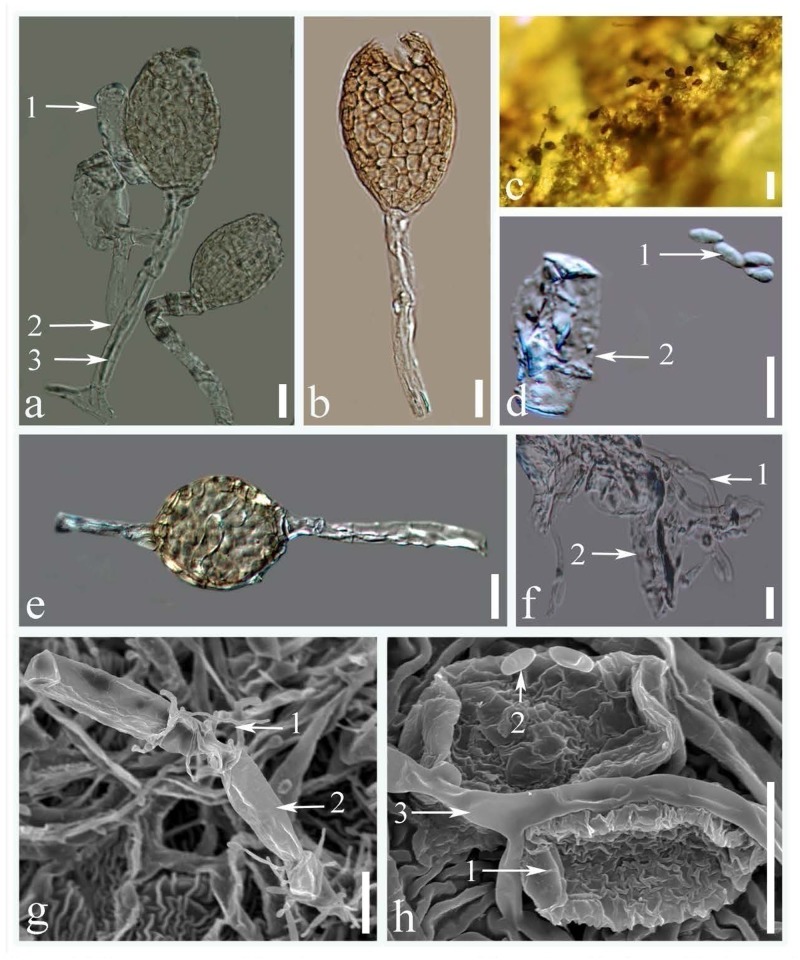
Microstructures of *Ampelomyces* spp.: **(a)** pycnidia produced in the conidiophores of rubber powdery mildews (1. conidia of rubber powdery mildew; 2. conidiophores of rubber powdery mildew; 3. intracellular hyphae of *Ampelomyces* spp.); **(b)** dehisced pycnidium by apical rupture; **(c)** pycnidium on the surface of a rubber leaf; **(d)** conidia (1. conidia of *Ampelomyces* spp.; 2. conidia of rubber powdery mildew); **(e)** pycnidia produced inside the hyphae of rubber powdery mildews; **(f)** superficial mycelium mat (1. mycelium of *Ampelomyces* spp.; 2. mycelium of rubber powdery mildew); **(g)** superficial growth of *Ampelomyces* mycelium on catanate type of conidia in rubber powdery mildew (1. mycelium of *Ampelomyces* spp.; 2. catanated conidiospore of rubber powdery mildew); **(h)** mycelium and spores of *Ampelomyces* spp. (1. non-catenate conidia (*Erysiphe quercicola*); 2. conidia of *Ampelomyces* spp., 3. mycelium of *Ampelomyces* spp.) (Scale bars: figures **a,b,d–h** = 10 μm, figure **c** = 20 μm).

All the leaf, flower and buds samples collected from 5 different sites in China were infected with *Ampelomyces* spp., and no infection was observed in the samples collected from other countries. Although *Ampelomyces* spp. has not previously been recorded as parasitizing rubber powdery mildew fungus, light microscopic (LM) observation of rubber powdery mildew colonies clearly showed that specimens collected from China were infected by *Ampelomyces* spp. (**Figures 1a–d**, **2c**). Mycelia of *Ampelomyces* spp. were superficial or immersed, hyaline, branched, septate (**Figures 2f,g**). Conidiomata were pycnidial and measured (32–49) × (19–32) μm (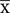 39.2 × 26.5μm, *n* = 30) with a 1.6–2.5 length/width ratio. They produced pycnidia intracellularly, in the hyphae, conidiophores and immature ascomata (**Figures 2a,b,e**). The pycnidia were separate, globose, or elongated to pyriform, unilocular, one-cell thick cell wall, of pale brown angular textured (**Figures 2a,b,e**). No distinct ostiole was present and dehisced by apical rupture of pycnidium. Conidiophores were absent. Conidia were (5–10) × (2.4–3.5) μm (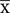 = 6.5 × 2.9 μm, *n* = 35) with a 2.1–4.1 length/width ratio. Conidia were very pale brown, aseptate, thin-walled, smooth, guttulate, straight, or curved, cylindrical to fusiform. (**Figures 2d,h**) Single-spore cultures grew slowly on PDA and took about 10–15 days, but sporulated profusely.

### Phylogenetic Analyses

**FIGURE 3:**
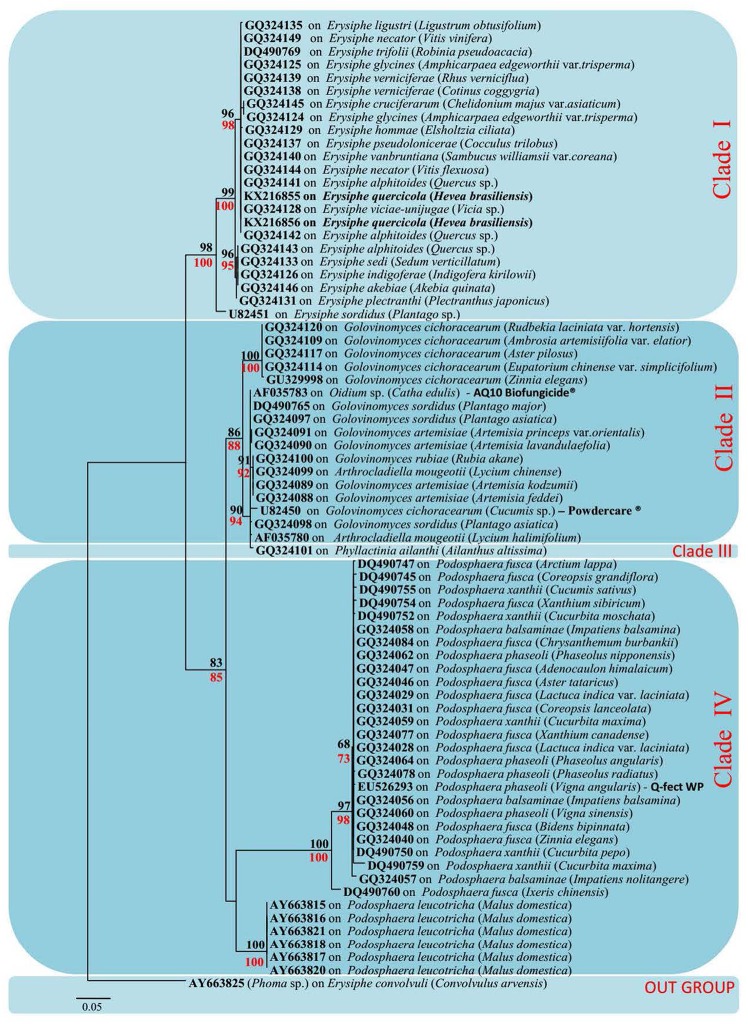
Maximum-likelihood (ML) tree based on a dataset of ITS sequences. Bootstrap support (BS) values for (ML, black) and maximum parsimony (MP, red) higher than 60% are defined as above the nodes. The tree is rooted to *Phoma* sp. (All mycohost species associated with *Ampelomyces* spp. are listed along with the GenBank accession number. The host plant for different mycohosts is presented in brackets. Sequences generated from this study are presented in bold text).

Due to the limited availability of sequence data for different genes in selected taxa in the GenBank, only ITS sequence data were used for these analyses. The data set was comprised of 73 sequences: 71 sequences retrieved from GenBank and 2 sequences from our samples infected with rubber powdery mildew (**Table 1**). To confirm the phylogenetic placement of *Ampelomyces* spp. identified in the present work, ML analyses and MP were carried out. This analyses comprised 512 total characters, of which 323 were constant, 133 were parsimony-informative and 56 parsimony-uninformative. One parsimonious tree was generated (**Figure 3**) and Bootstrap support (BS) values of MP (in red) and ML (in black) is shown on the upper branches (equal to or above 60% based on 1000 replicates). The Kishino–Hasegawa test showed that length = 300 steps with *CI* = 0.79, *RI* = 0.98, *RC* = 0.78, and *HI* = 0.21. The strains of *Ampelomyces* spp. on rubber powdery mildew grouped in Clade I, which includes different haplotypes infecting on *Erysiphe*, with relatively high (100%) bootstrap support (**Figure 3**).

**Table 1:** List of haplotypes with their host species, country of origin, and GenBank accession numbers.

Haplotype	No. of samples	GenBank accession no.	Host species	Country of origin
H1	15	GQ324145, GQ324124, GQ324139, GQ324138, GQ324137, GQ324140, GQ324141, GQ324142, GQ324144, GQ324149, DQ490769, GQ324125, GQ324128, **KX216855**, **KX216856**	*Erysiphe*	South Korea, China
H2	1	DQ490759	*Podosphaera*	China
H3	16	GQ324029, GQ324028, GQ324046, GQ324040, GQ324031, GQ324077, GQ324047, GQ324084, GQ324048, DQ490750, GQ324060, GQ324062, GQ324059, GQ324064, GQ324058, GQ324056	*Podosphaera*	South Korea
H4	1	DQ490747	*Podosphaera*	China
H5	1	DQ490754	*Podosphaera*	China
H6	1	DQ490760	*Podosphaera*	China
H7	1	DQ490745	*Podosphaera*	China
H8	1	DQ490755	*Podosphaera*	China
H9	1	DQ490752	*Podosphaera*	China
H10	1	GQ324078	*Podosphaera*	South Korea
H11	1	GQ324057	*Podosphaera*	South Korea
H12	5	AY663815, AY663818, AY663817, AY663816, AY663820	*Podosphaera*	Hungary, Germany, United Kingdom
H13	1	AY663821	*Podosphaera*	Hungary
H14	1	GU329998	*Golovinomyces*	South Korea
H15	4	GQ324114, GQ324117, GQ324120, GQ324109	*Golovinomyces*	South Korea
H16	4	GQ324100, GQ324088, GQ324089 GQ324099	*Golovinomyces*	South Korea
H17	2	GQ324091, GQ324090	*Golovinomyces*	South Korea
H18	3	GQ324097, DQ490765, AF035783	*Golovinomyces, Oidium* sp. (AQ10)	South Korea, Germany, Israel
H19	1	GQ324098	*Golovinomyces*,	South Korea
H20	1	GQ324129	*Erysiphe*	South Korea
H21	1	GQ324135	*Erysiphe*	South Korea
H22	1	AF035780	*Arthrocladiella*	Hungary
H23	1	GQ324101	*Phyllactinia*	South Korea
H24	1	U82451	*Golovinomyces*	Germany
H25	4	GQ324126, GQ324133, GQ324146, GQ324143	*Erysiphe*	South Korea
H26	1	GQ324131	*Erysiphe*	South Korea
H27	1	U82450	*Erysiphe*	Germany
H28	1	EU526293	*Sphaerotheca*	NA

*Sequences generated from this study are in bold text, NA, not available*.

### Haplotypes Identification

**FIGURE 4:**
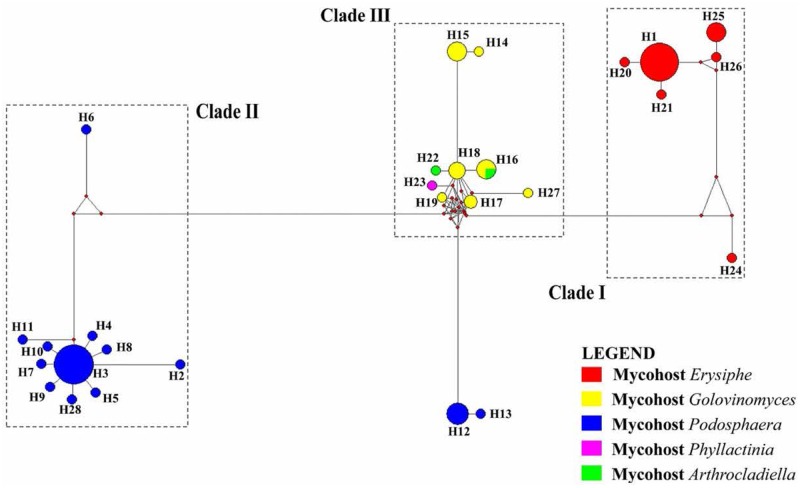
A network of 28 haplotypes of the ITS sequences of 74 *Ampelomyces* spp. from different mycohost genera. Clades are separated based on their mycohost. Lengths of the branches are proportional to the number of mutation points. The size of each circle is proportional to the frequency of that particular haplotype.

The alignment of 73 sequences of the ITS rDNA from *Ampelomyces* spp. were generated from the MUSCLE algorithm (Edgar, 2004). The ITS rDNA sequences of selected specimens from GenBank and two sequences from fresh isolates from rubber powdery mildew were 503 bp long and all contained 153 polymorphic sites. A total of 28 haplotypes (H1–H28) were identified with a very complex network of mutation events (**Table 1** and **Figure 4**).

## Discussion and Conclusion

In the present work, we identified for the first time *Ampelomyces* spp. parasitic on powdery mildew fungi of rubber. In the leaves samples collected, after 1–2 weeks incubation in growth chamber, the *Ampelomyces* spp. had devastated the powdery mildew colonies, leaving only the lesions created by the powdery mildews (**Figure 1**). This indicates that *Ampelomyces* spp. have the potentials to control rubber powdery mildew disease. However, further research on isolation, purification, and inoculation is required to confirm the biocontrol potentials of *Ampelomyces* spp. Furthermore, it was determined that the samples of leaves, inflorescences, and buds collected from China were found to be infected by *Ampelomyces* species. These results showed that the *Ampelomyces* spp. have much favorable weather conditions to propagate and devastate the rubber mildew in China as compared to the other countries. It could be the availability of *Ampelomyces* spp. spores in China as compared to other countries or may be the evolutionary behavior of the *Ampelomyces* spp. in China. LM and SEM analysis showed that most of the morphological features of *Ampelomyces* spp. were similar to other species that infect several distinct lineages of mycohost, but with some modification in the size, shape and color of pycnidia and spores. The mycelia of *Ampelomyces* spp. infecting rubber powdery mildews were hyaline, and the pycnidia and spores were pale brown in color. However, Lee et al. (2007) and Angeli et al. (2011) noted color variations in the mycelium and pycnidia from olive green to pale and dark brown in different mycohosts belonging to the genus *Erysiphe.* Pycnidia and spores were comparatively smaller in specimens of *Ampelomyces* isolated from rubber powdery mildews than in specimens isolated from other mycohosts in the same genus (Angeli et al., 2011).

The phylogenetic tree generated by our analyses contains four distinct Clades (**Figure 3**). Clade I includes all *Ampelomyces* spp. that infect *Erysiphe* spp. mycohosts. The specimens collected by the authors from rubber trees also clustered in Clade I with 100% bootstrap support. They cluster in Clade I because of the rubber powdery mildew also caused by a common fungal species *E. quercicola*, which belongs to genus *Erysiphe* (Limkaisang et al., 2005; Takamatsu et al., 2007). The majority of the mycohosts in Clade II belong to genus *Golovinomyces.* Some *Ampelomyces* spp. specimens, which infect fungi of genus *Arthrocladiella* also, clustered along with the genus *Golovinomyces*, indicating the similarities of their sequences to the mycoparasite in genus *Golovinomyces*. Although we used one specimen of *Ampelomyces* sp. that had infected the genus *Phyllactinia*, it is clustered separately in Clade III in the ML tree, indicating its genetic divergence. Clade IV includes all the *Ampelomyces* spp., which parasitizes *Podosphaera* spp. The clustering patterns of *Ampelomyces* spp. into different mycohost groups provide evidence for narrow host specialization within the *A. quisqualis* complex. *Ampelomyces* spp. are sometimes confused with *Phoma* spp., which are another pycnidial mycoparasites of powdery mildews. According to Kiss and some of his co-workers, *Phoma* spp. were fast growing and *Ampelomyces* spp. were slow-growing (Kiss, 1998; Kiss and Nakasone, 1998; Angeli et al., 2011). However, Kiss (1998) pointed out that these two taxa were not closely related and possibly not congeneric. Therefore, *Phoma* sp. (AY663825) was used as an outgroup taxon for this analysis.

The ML tree and haplotype network revealed some similar relationships of clustering pattern. The haplotypes H1, H20, H21, H24, H25, and H26 in network tree (Clade I) was similar to the clade 1 of the ML tree, and were associated with genus *Erysiphe.* In Clade I, H1 was the most dominant haplotype. The *Ampelomyces* spp. isolated from rubber powdery mildew also clustered in H1. This indicates that the sequence similarities of different isolates of *Ampelomyces* spp. to the rubber powdery mildew that cluster together in haplotype group H1. Remaining haplotypes of the Clade I in network tree was generated by creating mutation events of their sequences from the dominant haplotype H1. The haplotypes H2, H3, H5–H11, and H28 are in cluster IV in network tree also clustered similarly in Clade IV of MP tree belong the *Ampelomyces* spp. which was associated with *Podosphaera* sp. Haplotype H3 is the most dominant haplotype in this cluster and includes 16 specimens of *Ampelomyces* spp. used in this analysis. Similarly, haplotypes H14–H19, H22, H23, and H27 make a separate cluster in the haplotype network related to cluster II of the ML tree; this haplotype cluster includes *Ampelomyces* spp. associated with the mycohost group *Golovinomyces* spp. The phylogenetic tree also reveals that *Ampelomyces* spp. clusters together with mycohost *Arthrocladiella* spp. and *Golovinomyces* spp. The *Ampelomyces* spp. in mycohost *Arthrocladiella* spp. forms a sub-clade with *Golovinomyces* sp. and this indicates a series of mutation events have occurred. The *Ampelomyces* spp. in mycohost *Phyllactinia* was formed a sub-clade from mycohost *Golovinomyces* spp in network tree after having a one mutation event and therefore, cluster separately in clade III of ML tree. The haplotypes H12 and H13 were grouped separately in the network tree; in the ML tree, however, they grouped in the same cluster (Clade IV) along with the mycohost *Podosphaera* spp. This is because *Ampelomyces* spp. on haplotypes H12 and H13 are of European origin (**Table 1**) and the other haplotypes in clade IV are of Asian origin: mutations and geographic isolation has resulted in new speciation events.

After evaluating both the network tree and ML tree, haplotype analysis of ITS rDNA sequences revealed a distinct genetic lineage among specimens of *Ampelomyces* spp. These results further confirmed the conclusion of many previous authors (Lee et al., 2007; Park et al., 2010; Angeli et al., 2011). Although a single name is used for this species complex, *Ampelomyces* spp. is not in fact a single species. In order to address this problem, it will be necessary to conduct detailed morpho-molecular analyses with a large number of isolates associated with different mycohosts. The specimens isolated from rubber powdery mildew were clustered with the haplotype group H1 in the network tree with 13 other *Ampelomyces* spp. on mycohosts genus *Erysiphe*. This indicate that the possibility of use either one of the species in the haplotype group H1 as an effective microparasite for control the rubber powdery mildew. However, further research, including field trials, will be needed in order to confirm the effectiveness of these potential biocontrols. The commercial products “Q-fect” clustered in Clade IV with other *Podosphaera* spp. and “AQ10 Biofungicide^®^” and “Powdercare^®^”clustered in Clade II with mycohost genus *Golovinomyces* indicating that sequence similarities of the mycoparasite used to produce these commercial products to their particular genes. These results showed that these biopesticides are not ideal compound to control a wide range of powdery mildews. Identifying particular biocontrol agents which can target different powdery mildew groups is likely to bring rubber growers improved results in disease control. The further isolation, culturing, extraction, and investigation of the efficiency of different *Ampelomyces* species are essential to this effort. In addition, it will be necessary to perform both laboratory and field level experiments to investigate factors such as biocontrol application timing, inoculation potential, and the effect of environmental factors such as temperature and humidity on the growth of particular *Ampelomyces* sp. Such research could result in the development of a cost effective, environmentally safe alternative method of controlling the devastating rubber powdery mildew disease.

## Author Contributions

JX and KH conceived the original project and research plan. KL and SK designed and performed experiments. PM supervised most of the experiments. SB and SCK provide technical assistance. PM and SK analyzed the data and wrote the paper with contribution of all authors. All authors supervised and complemented the writing.

## Conflict of Interest Statement

The authors declare that the research was conducted in the absence of any commercial or financial relationships that could be construed as a potential conflict of interest. The reviewer CA and handling editor declared their shared affiliation.

## Footnotes

1 http://www.ncbi.nlm.nih.gov/
